# Diagnostic Accuracy of Platelet-Derived Parameters in Prognostication in Neurosurgery

**DOI:** 10.3390/ijerph19127115

**Published:** 2022-06-10

**Authors:** Michał P. Pluta, Magdalena Dziech, Tomasz Klocek, Anna J. Szczepańska, Łukasz J. Krzych

**Affiliations:** 1 Department of Anaesthesiology and Intensive Care, Faculty of Medical Sciences in Katowice, Medical University of Silesia, 40752 Katowice, Poland; a.j.szczepanska@gmail.com (A.J.S.); lkrzych@sum.edu.pl (Ł.J.K.); 2 Students’ Scientific Society, Department of Anaesthesiology and Intensive Care, Faculty of Medical Sciences in Katowice, Medical University of Silesia, 40752 Katowice, Poland; magdalena.dziech@gmail.com (M.D.); ttomek1996@gmail.com (T.K.)

**Keywords:** perioperative medicine, neurosurgery, risk, platelets

## Abstract

Introduction. Platelets (PLT) are key mediators in thrombotic and inflammatory processes. Their activity increases with size, so the mean platelet volume (MPV) can be a potential predictor of perioperative complications. The aim of the study was to assess the suitability of platelet parameters in predicting the risk of hospital death in neurosurgery. Methods. Retrospective observation covered 452 patients undergoing surgery in the period March 2018–August 2018. High-risk patients accounted for 44% (i.e., ASA-PS class III+) and 9% (i.e., ≥1 Shoemaker criterion), respectively, and 14% of procedures were performed in the urgent mode. The preoperative platelet parameters that were assessed and analysed were: total platelet count (PLT), mean platelet volume (MPV), plateletcrit (PCT) and platelet distribution width (PDW). The end point of the study was a hospital death. Results. Before discharge from the hospital, 13 patients died. The medians (IQR) PLT, MPV PDW and PCT were, respectively: 230 × 10^6^ L^−1^ (182–279); 9.2 fL (8.3–10.1); 14% (12.5–16.3); and 21% (17–26). PLT, PCT and PDW were not useful in the risk assessment. MPV was lower in patients who died (9.3 vs. 8.3 fL, *p* < 0.01) and predicted death occurred in 76% (AUC = 0.76, 95%CI 0.72–0.80, *p* < 0.01). Further, after adjustment for confounders, MPV remained a significant predictor of in-hospital death (logOR[MPV] = 0.31, AUC = 0.94, 95%CI 0.92–0.96, *p* = 0.02). Conclusion. The reduction in the average volume of platelets is associated with a worse prognosis in neurosurgical patients.

## 1. Introduction

Abnormal preoperative laboratory test results may support the identification of a narrow group of high-risk patients who generate more than 80% of perioperative complications [1]. Although it is likely that no single laboratory parameter will be a perfect predictor of perioperative risk, combining preoperative test results with available clinical data, including recognized prognostic scales, may increase diagnostic accuracy. Data from an analysis of complete blood count (CBC) may be a good example of this reasoning. We have previously shown increased accuracy in predicting complications in patients undergoing high-risk gastrointestinal surgery for the red blood cell distribution width (RDW) parameter [2]. Further, neutrophil-to-lymphocyte ratio in critically ill patients accurately predicted hospital death [3].

In the present study we decided to verify whether the values of basic platelet parameters that are determined by the automated CBC analyzer are useful in preoperative risk stratification in the neurosurgical patient population, and in combination with other perioperative risk assessment tools: the American Society of Anesthesiology scale (ASA-PS) and the Shoemaker criteria (SC) [4,5].

## 2. Materials and Methods

### 2.1. Study Design

A single-center, retrospective observational study was conducted between March 2018 and August 2018 at a university hospital in Poland. All consecutive 454 adult patients undergoing surgery in the Department of Neurosurgery who underwent CBC the day before surgery (for elective/accelerated surgery) or immediately before surgery (for urgent/emergency surgery) were included in the study. Patients with a diagnosis or suspected proliferative hematologic process were excluded from the final analysis (*n* = 2). None of the patients had blood product transfusions prior to neurosurgical intervention. There were no organ donors in the study group.

The results of the study were presented using the STROBE (Strengthening the Reporting of Observational Studies in Epidemiology) reporting guidelines [6].

Due to the observational, non-interventional nature of the project, the consent of the participants to participate in the study was not required (KNW/0022/KB/212/19) [7].

### 2.2. Laboratory Data

Blood was collected from a peripheral vein with a BD Vacutainer TM vacuum system (Becton Dickinson, Franklin Lakes, NJ, USA) into tubes containing edetainic acid (EDTA) and transferred to a local laboratory. A CBC analysis was performed automatically with a Sysmex XT-1800i hematology analyzer (Sysmex Corporation, Kobe, Japan) immediately after receiving the material. In terms of platelet parameters, the absolute platelet count (PLT), mean platelet volume (MPV), plateletcrit (PCT) and platelet distribution width (PDW) were determined.

### 2.3. Clinical Data

Patient data were collected, including basic demographics and previous medical history comprising diagnoses of anemia (Hb < 12 mg/dL for both genders); diabetes requiring insulin therapy; history of stroke or TIA; heart failure (according to European Society of Cardiology criteria [8]); pulmonary edema; respiratory failure; severe COPD; advanced vascular lesions involving the aorta; and sepsis (according to SCC 2016 criteria [9]). On this basis, individual patient risk was estimated using the ASA-PS and SC scales and defined as either low (ASA-PS I-II or none of the SC criteria met) or high (ASA-PS III-V or ≥1 SC met) [10]. The procedural risk for neurosurgical procedures was defined as moderate, based on guidelines from the European Society of Anesthesiology and Cardiology [11]. For an urgent or immediate procedure (ASA-PS “E”), the procedure risk was classified as high.

### 2.4. Outcome

The endpoint of the study was patient death before hospital discharge.

### 2.5. Statistical Analysis

The statistical analysis was performed using procedures that were available in MedCalc Statistical Software version 18.2.1 (MedCalc Software bvba, Ostend, Belgium; http://www.medcalc.org, accessed on 1 January 2022; 2018). The quantitative variables were presented as median and interquartile range (IQR). The qualitative variables were presented as absolute values and a percentage. The difference between the quantitative variables was assessed using an ANOVA or Kruskal–Wallis test, depending on the distribution of the variables. For the qualitative variables, a Chi-square test (*n* > 30) or Fisher’s exact test (*n* ≤ 30) was used, depending on the group size. The statistical association for qualitative variables was assessed by an odds ratio (OR) analysis along with 95% confidence intervals (CI). The diagnostic accuracy was assessed by ROC curves and area under curve (AUC). Finally, a logistic regression model was created in which the dependent variable was death before hospital discharge and the independent variables included ASA-PS classification, Shoemaker’s criteria, and preoperative MPV (all at *p* < 0.1 in simple analyses).

The criterion for statistical significance was *p* < 0.05.

## 3. Results

The study group included 452 patients. Individual risk was assessed as high for 44% and 9% of patients, respectively. Thirteen patients (3%) died before hospital discharge, including 9 patients undergoing urgent or immediate surgery (ASA-PS “E”). Baseline clinical and demographic data are shown in Table 1.

Patients who died before hospital discharge had significantly higher WBC and lower RBC, HGB, and MPV, compared to patients who survived (Table 2).

Considering SC, patients with advanced vascular disease involving the aorta had significantly lower MPV values compared to patients without vascular disease, respectively: 8.1 vs. 9.2 fL (*p* = 0.04). Regarding the other Shoemaker’s criteria, there were no significant differences in platelet parameters (*p* > 0.05 for all) (Table 3).

**Figure 1:**
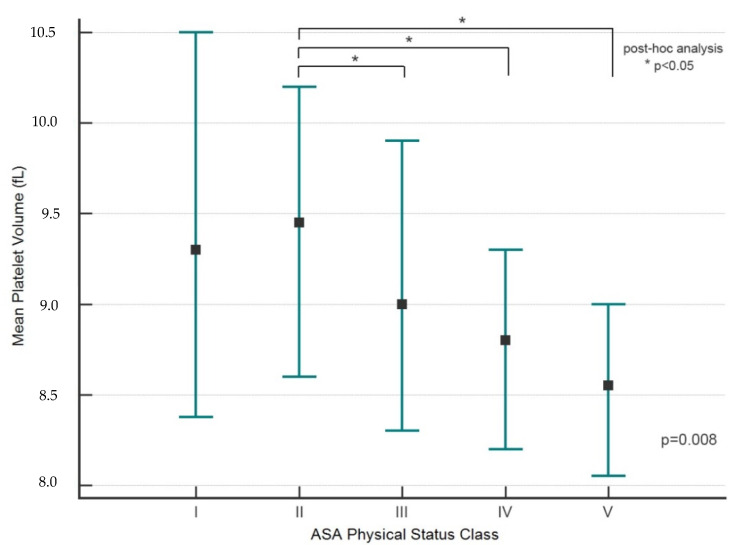
MPV and ASA-PS classification (post-hoc analysis). The marker indicates the median value (Me). The whiskers indicate an interquartile range (IQR, 25–75 pc); * *p* < 0.05 (post-hoc analysis).

As risk increased on the ASA-PS scale, patients had lower MPV values (*p* = 0.008) (Table 4). A post-hoc analysis showed significant differences in MPV values between grade II and ASA-PS grades III, IV and V (*p* < 0.05 for all) (Figure 1).

Patients whose individual risk was assessed as high according to ASA-PS and SC had significantly lower MPV values, respectively: 9.0 vs. 9.4 fL (*p* < 0.001) and 8.6 vs. 9.3 fL (*p* = 0.003) (Table 5).

There were higher preoperative PLT values (249, IQR 202–321 vs. 226 × 10^6^ L^−1^, IQR 180–276, *p* = 0.04) and lower MPV values (8.6, IQR 8.2–9.3 vs. 9.3 fL, IQR 8.4–10.2, *p* < 0.001) in the urgent or immediate surgery group (*n* = 61) compared to patients undergoing elective or accelerated surgery. There were no differences between the groups for PDW and PCT (*p* = 0.5 and *p* = 0.6, respectively).

**Figure 2:**
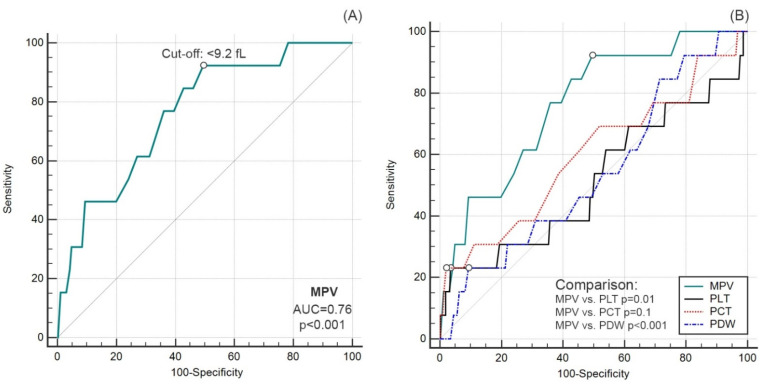
Diagnostic accuracy of MPV (**A**) and comparison of ROC curves for other platelet parameters (**B**).

Preoperative MPV ≤ 9.2 fL predicted death before hospital discharge with 76% accuracy (AUC = 0.76; 95%CI 0.72–0.80; *p* < 0.001), with a sensitivity of 92% and a specificity of 51% (Figure 2A). Predictive accuracy was not demonstrated for preoperative PLT, PDW and PCT values (*p* > 0.05 for all) (Figure 2B).

All patients who died before hospital discharge (*n* = 13) were categorized preoperatively as high individual risk according to the ASA-PS score. Patients classified into a higher category according to the ASA-PS had a greater than seven-fold increased risk of death (OR = 7.9, 95%CI 3.8–16.6). Of the 13 patients who died, 12 patients had preoperative MPV of ≤9.2 fL. An MPV of less than 1 fL was associated with a 12-fold increased risk of death (OR = 12.2, 95%CI 1.6–94.8). Based on Shoemaker’s criteria, five patients out of 13 patients with an unsuccessful outcome were classified preoperatively as high risk. Each additional risk factor increased the risk of death seven-fold (OR = 6.7, 95%CI 2.6–17.0) (Table 6).

The observations were finally confirmed in a logistic regression model. The MPV and ASA-PS score, independent of Shoemaker’s criteria, predicted in-hospital death. A 1 fL increase in MPV was associated with a 76% reduction in the risk of death (logOR = 0.31; 95%CI 0.13–0.73). Classification into a higher category on the ASA-PS score was associated with an eight-fold increased risk of death (logOR = 8.35; 95%CI 3.65–19.1). The predictive accuracy of the model was 94% (AUC = 0.94; 95%CI 0.92–0.96; *p* = 0.02) and was greater than the diagnostic accuracy of the ASA-PS score alone (AUC = 0.89; 95%CI 0.86–0.92, *p* < 0.001).

## 4. Discussion

In this retrospective observational study we demonstrated that combining preoperative MPV with an ASA-PS score increases the predictive accuracy of death before hospital discharge in the neurosurgical population.

CBC is routinely performed before most surgical procedures, but the benefit of this diagnostic strategy has not been proven [12]. Although Glance et al. in a study involving over 316,000 surgical patients demonstrated that thrombocytopenia found during routine preoperative testing was associated with a higher risk of transfusion and death [13], PLT count was not a useful predictive parameter of death in our study. Although performing routine coagulation tests before surgery has been shown to have no advantage over information from the patient’s medical history and physical examination [14], bleeding into the central nervous system structures is a strong risk factor for disability and an exponent of adverse neurological prognosis [15]. Limiting laboratory testing in neurosurgery to patients with significant medical history would save an estimated value of more than USD 80M each year [14]. This scientific dissonance calls for the individualization of management.

It remains unclear why abnormal MPV is associated with increased mortality and perioperative complications. Ensuring a normal PLT count is one of the prerequisites for maintaining hemostasis, but their proper function is more important than the absolute PLT count [16]. With the limited availability of advanced diagnostic tools, such as aggregometry or global hemostasis tests (thromboelastometry, thromboelastography), PLT function can be assessed indirectly using standard parameters that are determined in CBC [17]. Proinflammatory interleukins in response to injury stimulate the ejection of platelets stored in the spleen, which migrate to the site of tissue injury and become activated [18]. Elevated MPV correlates with the increased synthesis and release of thromboxane A2 and is therefore considered to be an indirect marker of thrombocyte aggregation [19]. It has been repeatedly shown that excessive PLT aggregation that is identified by elevated MPV is associated with a higher incidence of acute cardiac incidents [20]. Rzeplinski et. al. demonstrated a more than three-fold increased risk of adverse neurological outcome per 1 fL MPV in patients with subarachnoid hemorrhage in the setting of ruptured cerebral vascular aneurysm [21]. Among acute stroke patients, MPV was an independent predictor of adverse short-term outcome [22]. On the contrary, lower MPV may be a predictor of increased bleeding risk [23]. It cannot be ruled out that thromboembolic disease causes excessive PLT consumption, resulting in PLT that is released from the spleen having a reduced volume and therefore a lower MPV to maintain a constant platelet mass [17]. The conclusions of the above cited work support the reasonableness of our findings in the neurosurgical patient population.

In our study, there is a discrepancy in the identification of high-risk patients depending on the prediction tool that is used. All patients who died prior to discharge were originally classified as high risk according to the ASA-PS scale. When the Shoemaker criteria were used, only 38% of all hospital deaths qualified as high-risk. The difference results from the specificity of neurosurgical procedures and complications that determine the unfavorable prognosis. The analysis of deaths showed that postoperative mortality is mainly due to intracranial hemorrhage and intracranial hypertension, so the Shoemaker’s criteria may not be appropriate in this group of patients. Further research should address this problem and explain what predictive tools will accurately predict risks in neurosurgery.

Our study has several limitations. First, it was not designed to evaluate the causal relationship between MPV and the rate of hemorrhagic and thromboembolic complications, but the obtained results provide a basis for verifying these hypotheses in subsequent studies. Second, the study was of a retrospective nature, which also weakens the strength of scientific evidence. Third, the knowledge of the effect of antiplatelet drugs on MPV values is limited and comes from single reports [24]. This was also an argument not to include this factor in our analysis. In particular, antiplatelet therapy was routinely discontinued before surgery, according to current recommendations. Fourth, we did not analyze the time elapsed between the discontinuation of antiplatelet drugs and the determination of MPV in CBC.

## 5. Conclusions

Combining preoperative MPV assessment with anesthetic ASA-PS risk assessment can increase the accuracy of death prediction in neurosurgical patients. Our observations require confirmation in subsequent studies.

## Figures and Tables

**Table 1:** Baseline demographic and clinical data for the study population.

Variable	Value
Age [years]	
Me (IQR)	60 (46–68)
Female gender *n* (%)	229 (51%)
Shoemaker’s criteria	
Age > 70 years with evidence of limited reserve of one or more organs	22 (5%)
History of TIA/stroke	16 (4%)
History of heart failure/pulmonary edema/night dyspnea/bilateral auscultatory changes	16 (4%)
Respiratory failure	8 (2%)
Previous severe cardiorespiratory illness (acute myocardial infraction/stroke/severe COPD	10 (2%)
Advanced vascular diseases including aorta	4 (1%)
Sepsis	1 (<1%)
Shoemaker’s criteria: sum (*n*, %)	
No criterion met	411 (91%)
1 criterion	38 (8%)
2 criteria	3 (<1%)
Individual risk (according to SC), *n* (%)	
Low	411 (91%)
High	41 (9%)
ASA-PS (class)	
Median (IQR)	2 (2–3)
ASA-PS (class), *n* (%)	
I	37 (8%)
II	215 (47%)
III	166 (37%)
IV	26 (6%)
V	8 (2%)
Emergency mode (ASA-PS “E”)	63 (14%)
Individual risk (according to ASA-PS), *n* (%)	
Low	252 (56%)
High	200 (44%)
Type of surgery	
Ventricular drainage implantation	44 (10%)
Brain tumor, resection	108 (24%)
Aneurysm of the cerebral vessels, clipping	20 (4%)
Intracranial bleeding, craniotomy	45 (10%)
Brain edema, craniotomy	13 (3%)
Neuroinfection, brain abscess	1 (<1%)
Neuroinfection, ventricular drainage infection	2 (<1%)
Spine surgery	171 (37%)
Others	48 (11%)
Type of emergency surgery	
Intracranial bleeding, craniotomy	44 (10%)
Hydrocephalus, ventricular drainage implantation	3 (<1%)
Brain edema, craniotomy	13 (3%)
Neuroinfection, abscess brain drainage	1 (<1%)
Ventricular drainage infection	2 (<1%)
Outcome	
Death before hospital discharge, *n* (%)	13 (3%)
Cause of death	
Intracranial bleeding, brain edema	8 (2%)
Neuroinfection, septic shock	3 (<1%)
Ischemic stroke, cerebral edema	2 (<1%)

Me—median; IQR—interquartile range; BMI—body mass index; ASA-PS—American Society of Anesthesiologists Physical Status Classification System; SC—Shoemaker’s criteria; TIA—transient ischemic attack; COPD—chronic obstructive pulmonary disease.

**Table 2:** Selected peripheral blood morphological parameters evaluated preoperatively.

Parameter	All (*n* = 452) Me [IQR]	Survival (*n* = 439) Me [IQR]	Death (*n* = 13) Me [IQR]	*p*
WBC [×10^9^ L^−1^]	7.5 [6.0–10.1]	7.4 [5.9–9.7]	15.6 [10.2–24.9]	<0.001
RBC [×10^12^ L^−1^]	4.5 [4.1–4.9]	4.5 [4.1–4.9]	3.8 [3.1–4.7]	0.01
HGB [mg dL^−1^]	14.0 [12.9–15.1]	14.0 [12.9–15.1]	12.0 [9.4–14.7]	0.01
Hematocrit [%]	41 [38–45]	41 [38–45]	37 [29–43]	0.02
MCV [fL]	92 [88–95]	92 [88–95]	95 [89–99]	0.1
MCH [pg]	31 [30–32]	31 [30–32]	31 [29–34]	0.5
MCHC [g dL^−1^]	34 [33–34]	33 [33–34]	33 [33–34]	0.5
PLT [×10^6^ L^−1^]	230 [182–279]	230 [181–279]	230 [158–287]	0.8
PCT [%]	21 [17–26]	21 [17–26]	19 [13–25]	0.2
MPV [fL]	9.2 [8.3–10.1]	9.3 [8.4–10.1]	8.3 [7.6–8.8]	<0.001
PDW [%]	14.3 [12.5–16.3]	14.3 [12.5–16.3]	14.3 [11.7–15.8]	0.6

**Table 3:** Values of platelet parameters and Shoemaker’s criteria.

Shoemaker’s Criteria: Description	*n* (%)	PLT [×10^6^ L^−1^] Me (IQR)	*p*	MPV [fL] Me (IQR)	*p*	PCT [%] Me (IQR)	*p*	PDW [%] Me (IQR)	*p*
Previous acute MI/stroke/severe COPD	Yes: 10 (2%) No: 442 (98%)	193 (168–209) 231 (182–279)	0.07	8.6 (7.9–9.2) 9.2 (8.4–10.1)	0.1	17 (14–22) 21 (17–26)	0.1	15 (13–18) 14 (12–16)	0.4
Age > 70 years with evidence of limited reserve of one or more organs	Yes: 22 (5%) No: 432 (95%)	213 (151–266) 231 (183–279)	0.1	8.8 (8.2–10.1) 9.2 (8.3–10.1)	0.5	19 (15–23) 21 (18–26)	0.1	14 (12–18) 14 (13–16)	0.9
Advanced vascular diseases including aorta	Yes: 4 (1%) No: 448 (99%)	274 (213–332) 230 (181–279)	0.3	8.1 (7.7–8.7) 9.2 (8.3–10.1)	0.04	22 (19–26) 21 (17–26)	0.8	13 (11–14) 14 (13–16)	0.2
Sepsis	Yes: 1 (<1%) No: 451 (99%)	401 (403–403) 230 (181–279)	0.1	7.0 (7.0–7.0) 9.2 (8.3–10.1)	0.1	28 (28–28) 21 (17–26)	0.3	10 (10–10) 14 (13–16)	0.1
Respiratory failure	Yes: 8 (2%) No: 444 (98%)	248 (217–286) 229 (181–279)	0.5	8.5 (8.1–8.9) 9.2 (8.3–10.1)	0.1	21 (18–26) 21 (17–26)	1.0	14 (13–16) 14 (12–16)	1.0

Me—median; IQR—interquartile range; MI—myocardial infraction; COPD—chronic obstructive pulmonary disease.

**Table 4:** Platelet parameter values and ASA-PS classification.

Parameter	ASA-PS Me (IQR)					*p*
	I	II	III	IV	V	
PLT [×10^6^ L^−1^]	244 (194–283)	236 (183–279)	219 (181–279)	218 (151–281)	240 (172–300)	0.8
PCT [%]	21 (19–27)	22 (18–26)	20 (17–25)	20 (15–24)	21 (15–26)	0.3
PDW [%]	14 (12–16)	14 (13–17)	14 (12–16)	14 (13–16)	15 (14–17)	0.6
MPV [fL]	9.3 (8.4–10.5)	9.5 (8.6–10.2)	9.0 (8.3–9.9)	8.8 (8.2–9.3)	8.6 (8.1–9.0)	0.008

Me—median; IQR—interquartile range; ASA-PS—American Society of Anesthesiologists Physical Status Classification System.

**Table 5:** PLT and MPV values, and individual risk according to ASA-PS and SC.

Parameter	ASA-PS Me (IQR)		*p*	Shoemaker’s Criteria Me (IQR)		*p*
	Low Risk (ASA I–II)	High Risk (ASA III–V)		Low Risk	High Risk (≥1 Criterion)	
PLT [×10^6^ L^−1^]	236 (184–279)	222 (181–279)	0.2	231 (183–279)	220 (168–285)	0.4
PCT [%]	22 (18–26)	20 (17–25)	0.1	21 (18–26)	20 (15–23)	0.1
PDW [%]	14.3 (12.5–16.8)	14.3 (12.2–15.9)	0.3	14.3 (12.5–16.3)	14.1 (12.1–16.2)	0.7
MPV [fL]	9.4 (8.4–10.3)	9.0 (8.3–9.2)	<0.001	9.3 (8.4–10.1)	8.6 (7.8–9.3)	0.003

Me—median; IQR—interquartile range; ASA-PS—American Society of Anesthesiologists Physical Status Classification System.

**Table 6:** Individual risk and treatment outcome.

Individual Risk	Survival *n* (%)	Death *n* (%)	*p*
ASA-PS			
Low (ASA-PS I–II) (*n* = 252)	252 (56%)	-	0.04
High (ASA-PS III–V) (*n* = 200)	187 (41%)	13 (3%)	
Shoemaker’s criteria			
Low (none of the criteria met) (*n* = 411)	403 (89%)	8 (2%)	0.4
High (≥1 criterion met) (*n* = 41)	36 (8%)	5 (1%)	
MPV ^1^			
≤9.2 fL (*n* = 226)	214 (47%)	12 (3%)	0.002
>9.2 fL (*n* = 226)	225 (50%)	1 (<1%)	

ASA-PS—American Society of Anesthesiologists Physical Status Classification System; ^1^ the cut-off value for MPV was based on the AUC analysis.

## Data Availability

Data are available from the authors of the study.
